# Training Convolutional Neural Networks with Multi-Size Images and Triplet Loss for Remote Sensing Scene Classification

**DOI:** 10.3390/s20041188

**Published:** 2020-02-21

**Authors:** Jianming Zhang, Chaoquan Lu, Jin Wang, Xiao-Guang Yue, Se-Jung Lim, Zafer Al-Makhadmeh, Amr Tolba

**Affiliations:** 1 Hunan Provincial Key Laboratory of Intelligent Processing of Big Data on Transportation, School of Computer and Communication Engineering, Changsha University of Science and Technology, Changsha 410114, China; jmzhang@csust.edu.cn (J.Z.); lcq@stu.csust.edu.cn (C.L.); jinwang@csust.edu.cn (J.W.); 2 School of Information Science and Engineering, Fujian University of Technology, Fuzhou 350118, China; 3 Rattanakosin International College of Creative Entrepreneurship, Rajamangala University of Technology Rattanakosin, Nakhon Pathom 73170, Thailand; x.yue@external.euc.ac.cy; 4 Department of Computer Science and Engineering, School of Sciences, European University Cyprus, Nicosia 1516, Cyprus; 5 Liberal Arts & Convergence Studies, Honam University, Gwangju 62399, Korea; 6 Computer Science Department, Community College, King Saud University, Riyadh 11437, Saudi Arabia; zalmakhadmee@ksu.edu.sa (Z.A.-M.); atolba@ksu.edu.sa (A.T.); 7 Mathematics and Computer Science Department, Faculty of Science, Menoufia University, Shebin-El-kom 32511, Egypt

**Keywords:** dropout, triplet loss, training with multi-size images, remote sensing scene classification

## Abstract

Many remote sensing scene classification algorithms improve their classification accuracy by additional modules, which increases the parameters and computing overhead of the model at the inference stage. In this paper, we explore how to improve the classification accuracy of the model without adding modules at the inference stage. First, we propose a network training strategy of training with multi-size images. Then, we introduce more supervision information by triplet loss and design a branch for the triplet loss. In addition, dropout is introduced between the feature extractor and the classifier to avoid over-fitting. These modules only work at the training stage and will not bring about the increase in model parameters at the inference stage. We use Resnet18 as the baseline and add the three modules to the baseline. We perform experiments on three datasets: *AID*, *NWPU-RESISC45*, and *OPTIMAL*. Experimental results show that our model combined with the three modules is more competitive than many existing classification algorithms. In addition, ablation experiments on *OPTIMAL* show that dropout, triplet loss, and training with multi-size images improve the overall accuracy of the model on the test set by 0.53%, 0.38%, and 0.7%, respectively. The combination of the three modules improves the overall accuracy of the model by 1.61%. It can be seen that the three modules can improve the classification accuracy of the model without increasing model parameters at the inference stage, and training with multi-size images brings a greater gain in accuracy than the other two modules, but the combination of the three modules will be better.

## 1. Introduction

### 1.1. Background

Remote sensing scene classification intends to classify an image into various semantic categories by directly modeling the scenes by exploiting the variations in spatial arrangements and structural patterns [1]. Remote sensing images have many categories and complicated spatial information, which make it a challenging task to effectively describe and classify remote sensing images [2]. Remote sensing images play an important role in military, civil engineering, agriculture, and other fields [2]. With the further development of remote sensing equipment and wireless sensor networks [3,4,5,6], the way of obtaining remote sensing images is more convenient, and the demand for remote sensing scene classification is getting increasingly more urgent. Therefore, many researchers pay attention to this field [1,2,7,8,9,10,11,12]. At present, many researchers use neural network technology to model remote sensing images and learn a nonlinear input–output mapping model from massive remote sensing data, thus realizing an automatic classification system of remote sensing scenes [1,2,13].

Currently, the methods of remote sensing image scene classification are mainly classified into three classes based on low-level visual features, middle-level visual features, and high-level visual features. Low-level visual features include the scale-invariant feature transform (SIFT) [7], histogram of oriented gradients (HOG) [8], gray-level co-occurrence matrix (GLCM), etc. The low-level visual features based methods take low-level visual features as descriptors of remote sensing images [1]. Generally, it is difficult for a single low-level visual feature to fully describe a scene, so researchers combine multiple low-level visual features to enhance the classification accuracy of the model [9]. Those methods based on low-level visual features achieve well on some remote sensing scenes with uniform structures and spatial arrangements, but it is difficult for them to depict the high diversity and nonhomogeneous spatial distributions in remote sensing scenes [7]. Unlike the low-level visual features based methods, the middle-level visual features based methods first extract local image features, such as SIFT and local binary pattern (LBP), and then encode these features to construct an overall middle-level representation for remote sensing images [1]. Bag-of-visual-words (BoVW)-based methods are the most common of this kind of method [14,15,16,17]. For these BoVW-based methods, various hand-crafted local image descriptors are used to represent remote sensing scenes [1]. One main difficulty of such methods lies in the fact that they may lack the flexibility and adaptivity to different scenes [1]. Compared to the middle-level visual features based methods, the high-level visual features based methods have a better classification performance. Compared to middle-level visual features and low-level visual features, the features extracted by the convolution neural network (CNN) are more abstract and discriminative [1]. In the early research, many researchers used remote sensing datasets to directly train the network. Later, researchers found that the model pre-trained on ImageNet can obtain a better classification performance [2]. Some researchers use a support vector machine (SVM) [18] as a classifier to classify the features extracted by the neural network [19,20]. Unlike using SVM, an end-to-end model can be constructed by using a fully connected layer for classification [2,13].

### 1.2. Motivation

CNN is widely used in remote sensing scene classification [21,22,23] because of its powerful feature extraction capability in various fields such as object tracking [24,25,26], detection [27], and classification [28]. Compared to fine-tuning existing models [29], many researchers focus on the design of the network model [2,13,30], aiming to obtain a higher classification accuracy via additional modules. For example [13], in addition to using Resnet [31] to extract feature maps from the image, Zhang et al. [13] designed a convolutional network to extract feature maps from attention maps [32]. These feature maps will be fused with the feature maps extracted by Resnet for classification. From these works [2,13,30], we find that the additional modules used at the inference stage bring about a performance improvement, but also bring about more parameters and complexity [33,34,35], which may be unfavorable for deployment on devices with limited memory and computational resources [36]. Therefore, it is necessary to explore how to improve the classification performance of the model without increasing the parameters.

Some works show that the diversity of data can affect the classification accuracy of the model [37,38]. However, many models do not accept multi-size images as the input [1,2,13], which leads to the lack of data diversity. To address this problem, we embed adaptive pooling. Under the effect of adaptive pooling, feature maps are sampled to a fixed size so that the model can process images with different sizes. After that, we scale the image at multiple scales and propose a multi-size image training strategy to ensure the diversity of images. It should be noted that our strategy only exists at the training stage and will not increase model parameters at the inference stage.

Some works only improve the classification performance of the model by embedding additional modules [2,13] but ignore intra-class diversity and inter-class similarity. In order to alleviate this problem, we introduce more supervision information to guide the model to learn better features. Inspired by FaceNet [39], which aims to learn a highly discriminative face feature via triplet loss, we introduce triplet loss to guide the model to learn a more discriminative remote sensing scene feature. In addition, we specially design a branch for triple loss.

Too many parameters make the network obtain good performance on the training set by memorizing the data, but the performance on the test set is very poor. The lack of generalization ability leads to overfitting of the network. Generally, neural networks have a large number of parameters. Therefore, overfitting is a serious problem in such networks [40]. To address this problem, dropout [40], an avoiding overfitting technique, is applied to various models [30,40]. Dropout reduces the connection between neurons by randomly discarding neurons, thus weakening the correlation between neurons. The use of dropout will increase the training time [40]. Unlike [30] using multiple dropouts, we only use one dropout between the feature extractor and the classifier.

### 1.3. Main Contributions

Generally, the main contributions of our work are as follows:

1) We specially design a strategy to train the network, namely, training with multi-size images. An adaptive pooling is embedded for fixing the size of the feature maps, which makes the model process images with different sizes, enabling training on images with different sizes.
2) A branch dedicated to triplet loss is designed. The main purpose of this branch is to guide the model to learn a more discriminative feature vector of remote sensing scenes, thus improving the classification accuracy of the model.
3) The dropout technology is utilized between the feature extractor and classifier to avoid overfitting, therefore refining the generalization capability of the model.
4) Wintegrate the three modules mentioned above into the model to improve the classification accuracy without increasing parameters of model at the inference stage, and the end-to-end model that we constructed can classify the remote sensing scene well.

## 2. Materials and Methods

### 2.1. Materials

#### 2.1.1. Dataset

**Figure 1:**
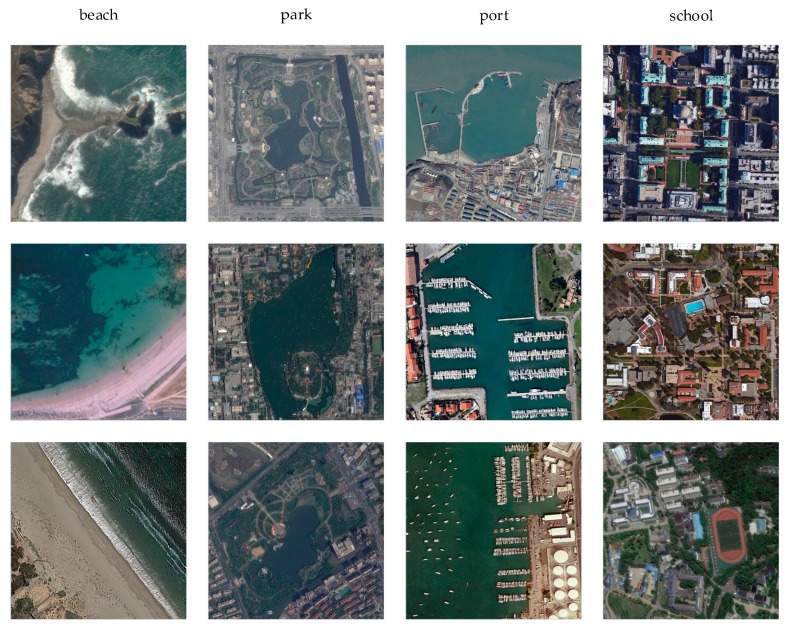
Some examples of remote sensing scene images. They are beach, parks, port, and school.

We perform experiments on three commonly used datasets, and some image samples are shown in Figure 1. Information about the three datasets is shown in Table 1. These datasets are as follows.

Aerial image dataset (*AID*) [1]. *AID* includes the following scenes: Industrial, parking, dense residential, mountain, railway station, bridge, center, storage tanks, church, pond, viaduct, baseball field, stadium, port, bare land, forest, school, desert, square, river, resort, sparse residential, commercial, playground, meadow, park, airport, farmland, medium residential, and beach.

A benchmark created by Northwestern Polytechnical University for remote sensing image scene classification (*NWPU-RESISC45*) [41]. *NWPU-RESISC45* includes the following scenes: Mountain, basketball court, sparse residential, cloud, bridge, ship, medium residential, meadow, chaparral, sea ice, palace, railway station, golf course, storage tank, snowberg, forest, lake, overpass, beach, thermal power station, stadium, roundabout, church, mobile home park, commercial area, ground track field, parking lot, baseball diamond, freeway, wetland, desert, airplane, island, railway, industrial area, airport, terrace, tennis court, rectangular farmland, runway, dense residential, harbor, river, intersection, and circular farmland.

A benchmark created by the center for optical imagery analysis and learning (*OPTIMAL*) [2]. *OPTIMAL* includes the following scenes: Overpass, mountain, round farmland, desert, runway, roundabout, freeway, forest, business district, square farmland, meadow, crossroads, bridge, parking lot, harbor, airport, island, bushes, beach, factory, medium houses, railway, mobile house area, church, baseball field, dense houses, playground, basketball court, golf field, lake, and airplane.

In order to effectively evaluate the performance of the model, we perform five experiments on three datasets. Details of the partition ratio of datasets are shown in Table 2.

#### 2.1.2. Experimental Environment

All experiments are performed on an Ubuntu 16.04.5 LTS, and the deep learning framework used is pytorch1.3.0. The CPU of the server is Intel(R) Xeon(R) CPU E5-2640 v4 @ 2.40 GHz, and the GPU is a GeForce GTX 1080TI. The driver version of the GPU is 430.40, and the version of CUDA is 10.1.

### 2.2. Methods

#### 2.2.1. Overall Framework

Our overall framework is divided into two parts: Feature extractor *f* and classifier *Classifier*.

(1) F=f(X),

(2) $$C_{F}=Classifer(F),\;$$

(3) $$O_{F}=softmax(C_{F}).\;$$

**Figure 2:**
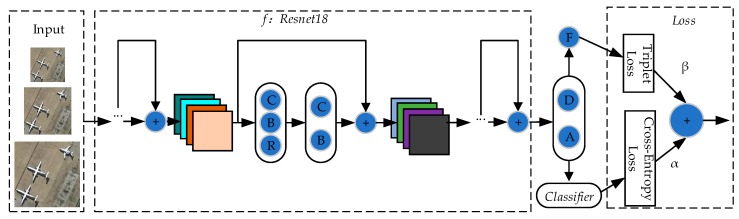
Overall framework. **C** represents convolution, **B** represents BatchNorm [42], **R** represents activation function ReLU [43], and “**+**” represents element-wise additions. **D** represents dropout. **A** represents adaptive pooling. **F** represents the fully connected layer, which serves Triplet Loss. α is the weight of Cross-Entropy Loss, and β is the weight of Triplet Loss.

The feature extractor *f* extracts the features from image *X* to obtain feature *F* ($F\in R^{1\times M}$, *M* is defined as the length of a feature. In our experiment, we set *M* = 512). Then, the feature *F* is classified by *Classifier* to obtain classification result $C_{F}$ ($C_{F}\in R^{1\times N}$, *N* is defined as the number of categories). The category probability $O_{F}$ ($O_{F}\in R^{1\times N}$) is obtained after *softmax*. The category with the highest probability is the final classification result. The overall framework is shown in Figure 2. More details will be given in Section 2.2.2, Section 2.2.3, Section 2.2.4, Section 2.2.5, Section 2.2.6 and Section 2.2.7.

#### 2.2.2. Feature Extractor *f*

As mentioned above, the quality of a feature directly affects classification accuracy. Many classic networks have excellent feature extraction capabilities, such as AlexNet [44] and VGGNet [45]. Empirically, deeper network structures can extract feature maps with more semantic information. Compared to AlexNet and VGGNet, Resnet [31] has a deeper depth. In addition, Resnet can greatly alleviate the problem of network degradation [31]. Therefore, we choose Resnet18 [31] as the feature extractor to extract features from images. Resnet18 mainly consists of residual blocks, which are composed of stacked convolutional layers and cross-layer connection. The cross-layer connection shortens the distance between non-adjacent layers so that the gradient can be better for back-propagation. On the other hand, it enables the network to automatically learn the path of feature movement. That is to say, once a deeper network path is found to be a better choice, the path will be executed. On the contrary, if the deep path is found to be unsuitable, the path is directly ignored, and the short path is selected, that is, the original feature maps are maintained, which will not affect the performance of the network.

#### 2.2.3. Classifier

The *Classifier* consists of a fully connected layer. The input dimension of this *Classifier* is 512, and the output dimension is determined by the number of categories. For example, on the *AID* dataset containing 30 categories, the output dimension of the classifier is set to 30.

#### 2.2.4. Dropout

**Figure 3:**
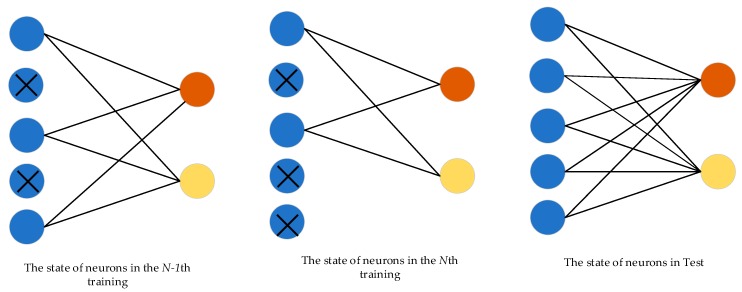
Working principle of dropout. “×” means that the neuron is dropped out.

Generally, neural networks have a large number of parameters. However, overfitting is a serious problem in such networks. To alleviate this problem, a dropout layer [40] is used between the feature extractor and the classifier. The working principle is shown in Figure 3. Neurons of the feature extractor are dropped out with a certain probability. This means that a new network with a few parameters is trained in each training. When training is complete, dropout is closed and no longer works. At this time, the classification results of the model are the overall results of all the trained networks, which is similar to the model integration. We know that model integration tends to obtain better results than the single model. From another point of view, dropout breaks the connection between some features and classifiers during training, which weakens the joint adaptability between features and increases the generalization capability of the model.

#### 2.2.5. Triplet Loss

The features of remote sensing images from different categories may be very similar, while the features of images from the same category may have large differences. Although CNN has an excellent feature extraction capability, the features extracted by CNN are not effectively used for classification, because of the existence of intra-class diversity and inter-class similarity. To alleviate this problem, we utilize triplet loss to guide the learning of CNN, which is proposed in [39] for the first time to learn a better face embedding. Triple loss is suitable for shortening the distance between images of the same category and widening the distance between images of different categories. The triplet loss formula can be expressed as follows:

(4) $$L_{t}=max(d(a,p)-d(a,n)+margin,0).$$

**Figure 4:**
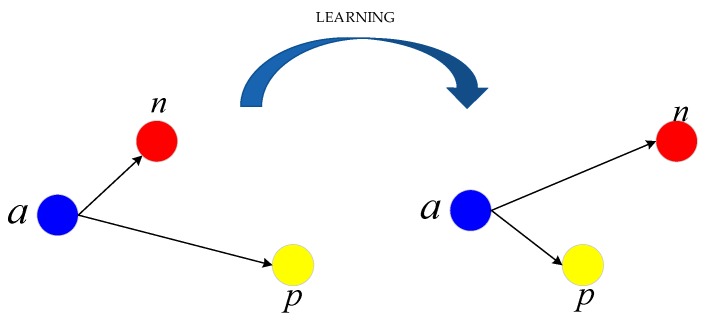
Triplet loss guides model learning with more discriminative features during the training process.

In Equation (4), *a* represents a feature vector of an image, *p* represents a feature vector of an image from the same category, and *n* represents a feature vector of an image of a different category from *a*. *d* represents the Euclidean distance of two vectors. *margin* is the threshold between positive and negative sample pairs, which controls the generation of supervision information. In our experiments, *margin* is set to 1.4. From Equation (4), we can find that the smaller *d*(*a*,*p*) is, the larger *d*(*a*,*n*) is, and the smaller $L_{t}$ is. That means that triple loss can guide the model to shorten the distance between images of the same category and extend the distance between images of different categories, as shown in Figure 4.

For convenience, we define (*a*,*p*) as a positive sample pair and (*a*,*n*) as a negative sample pair. From Equation (4), it can be known that triplet loss needs samples such as (*a*,*p*,*n*) to be the input. According to the rules of the training sample generation, the training of triplet loss can be classified into two types: Offline training and online training. For offline training, the training samples are generated before training. More specifically, three images are extracted from the image set, two of which belong to the same category, and the remaining image belongs to another category, which can be expressed as (*a*,*p*,*n*). According to this rule, a large number of input samples can be generated. It should be noted that most of the generated samples can meet the condition *d*(*a*,*p*) − *d*(*a*,*n*) < 0, which means that most samples cannot provide loss and the model cannot be further optimized. For online training, the samples (*a*,*p*,*n*) are constructed by using the image vectors of the current training batch. In this paper, we adopt the online training strategy. In order to generate more effective training samples, we adopt the batch hard triplet loss algorithm. The algorithm is shown in Algorithm 1, which is written in python and pytorch style.
**Algorithm 1** Batch hard triplet loss**INPUT**: *labels*, *embeddings*, *margin**labels*: the labels of images. *labels*$\in R^{B}$, *B* represents the number of images of a training batch
*embeddings*: the feature vectors of images, *embeddings*$\in R^{B\times M}$, *M* represents the length of the feature vectors of the images.**OUTPUT:** triplet_loss**1.** Calculate the European distance *distances* between every two embeddings in *embeddings*, *distances*
$\in R^{B\times B}$
  ***distances* =**
***calculate_pairwise_distances*(*embeddings*)****2.** Calculate the mask of the same category, which is marked as *mask_positive*. *mask_positive* is a matrix of B×B, which only contains 0 or 1. It is used to select distances of the same category.
  ***mask_positive* =**
***calculate_valid_positive_mask*(*labels*)****3.** Calculate the mask of the different category, which is marked as *mask_negative*, *mask_negative* is a matrix of B×B, which only contains 0 or 1. It is used to select distances of the different category.
  ***mask_positive* =**
***calculate_valid_negative_mask***
**(*labels*)****4.** Calculate the distance of hard positive sample pairs, which is marked as *hardest_positive_dist*. *hardest_positive_dist*
$\in R^{B\times 1}$.
  ***hardest_positive_dist* =**
(distances * mask_positive.float()).*max*(*dim=1*)[*0*]**5.** Calculate the distance of hard negative sample pairs, which is marked as *hardest_negative_dist*. *hardest_negative_dist*
$\in R^{B\times 1}$.
  ***max_dist* =**
***distances.max*(*dim=1, keepdim=True*)[*0*]**  ***distances* =**
***distances*+**
max_dist * (*mask_negative.eq*(*0*)*.byte*()).***float*()**  ***hardest_negative_dist*** = ***distances.min*(*dim=1*)[*0*]****6.** Calculate triplet loss, which is marked as *triplet_loss*. *triplet_loss*
∈R.
  ***triplet_loss*=(*hardest_positive_dist-hardest_negative_dist+margin*)*.clamp*(*min=0*)**  ***triplet_loss* =**
***triplet_loss.mean*()**  ***return triplet_loss***

It should be noted that if the feature vector extracted by the feature extractor is directly used to calculate triplet loss, the feature vector with large dimensions will lead to instability of triplet loss. In addition, triplet loss will directly affect the feature extractor, which may have side effects on cross-entropy loss. To this end, we design a fully connected layer to reduce the dimension of the feature vector. The feature vectors after dimensionality reduction are used to calculate triplet loss. It is worth noting that the branch we designed is removable, that is to say, the branch participates in the adjustment of model parameters during training, and is removed after training. In this way, the number of parameters and the computing overhead of the model at the inference stage are not increased, but the gain of accuracy brought by triplet loss is obtained.

#### 2.2.6. Training with Multi-Size Images

In general, the lengths of the feature vectors extracted from images with different sizes are not the same. Due to this limitation, only images with a single size can be used for training CNNs, which leads to the lack of data diversity. In order to allow the model to process images with different sizes, we modify the feature extractor. More specifically, inspired by [38], we introduce adaptive pooling, which replaces the last pooling of the Resnet18. The adaptive pooling fixes the size of the final features to 512 × 1 × 1. That is, as long as the input image size is larger than 224 × 224, the dimensions of the features extracted by the extractor are fixed to the size of 512 × 1 × 1. There are two strategies for training models using images with different sizes. One is that the image size is generated randomly in each training epoch. For this strategy, the scale transform present in the two epochs before and after may be relatively large, resulting in large training fluctuations, which increases the difficulty of training. The other is the slow scale transform from a small size to a large size. In this way, the image size used by the two epochs before and after is not much different. The image size used by the last epoch is significantly different from the image size used by the first epoch, which makes the model learn a more versatile “knowledge.” In this paper, we adopt the second strategy. More specifically, the model is trained for 10 epochs using images with the size of 224 × 224 first; then, the model is trained for 10 epochs using images with the size of 256 × 256; after that, the model is trained for 10 epochs using images with the size of 288 × 288; finally, the model is trained for 50 epochs using images with the size of 320 × 320.

#### 2.2.7. Loss

We combine cross-entropy loss and triplet loss as the final loss. The cross-entropy loss is described as follows:

(5) $$L_{c}=-\frac{1}{B}*\overset{B}{\underset{i=1}{\sum }}log\frac{e^{W_{y_{{}_{i}}}^{T}x_{i}+b_{y_{{}_{i}}}}}{\overset{n}{\underset{j=1}{\sum }}e^{W_{j}^{T}x_{i}+b_{j}}}.$$

$x_{i}$ represents the feature vector extracted by the extractor, and *W* and *b* represent the parameters of *Classifier*, which consists of a fully connected layer. $y_{i}$ indicates the category of the *i-*th image. The final loss is

(6) $$L=\alpha L_{c}+\beta L_{t}.$$

In our experiments, α is set to 1 and β is set to 0.5.

### 2.3. Evaluation Metrics

There are many metrics that can evaluate the classification performance of the model, such as quantity disagreement [46], allocation disagreement [46], overall accuracy [47,48,49,50,51], and confusion matrix. At present, many researchers use the overall accuracy and confusion matrix to evaluate the model in the field of remote sensing scene classification [1,2,13,21,22,23]. In order to easily compare our model with the existing algorithms, we also use the overall accuracy and confusion matrix as evaluation metrics.

Overall accuracy (OA) is the ratio of the number of samples correctly predicted by the model on the test set to the total number of samples.

A confusion matrix is a specific matrix that is used to visualize the performance of a model. In this matrix, each row represents the actual categories and each column represents the predicted value [2].

### 2.4. Hyper Parameters

In our experiment, Adam, a gradient descent optimization algorithm, is used for optimizing the parameters of the model, the learning rate is set to 1e–4, and the weight decay rate is set to 5e–4. As mentioned above, to calculate triplet loss, we reduce the dimensions of the features extracted by the extractor. We set the length of the feature vector for calculating triplet loss to 128. The parameter margin of triplet loss is set to 1.4. The weight β of triplet loss is set to 0.5, and the weight α of cross-entropy loss is set to 1. The batch size is set to 128. The performance of the model is evaluated every 2 epochs on the validation set. The weights of the model with the best performance is saved after the training of 80 epochs. In addition, the learning rate decays every 32 epochs, and the decay rate is 0.5.

## 3. Results and Discussion

In this section, we show the classification performance of the algorithm integrating the three modules on *AID* and *NWPU-RESISC45* datasets and the comparison results with some existing algorithms. After that, we show the ablation experiments of each module.

### 3.1. Experimental Results on AID Dataset

In this section, we show the classification performance of our model on the *AID* dataset and the comparison results with some existing algorithms.

**Figure 5:**
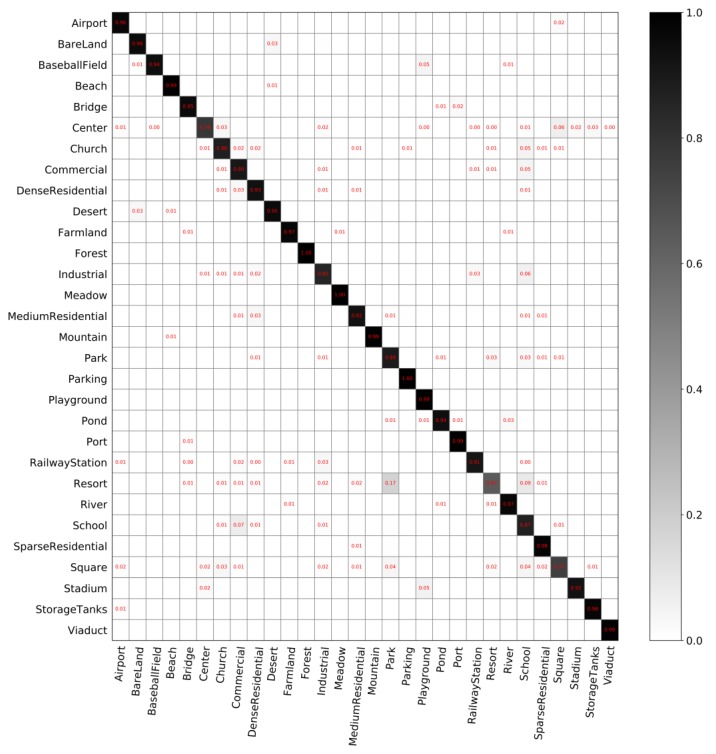
Confusion matrix on *AID* with training set of 10% and validation set of 10%.

**Figure 6:**
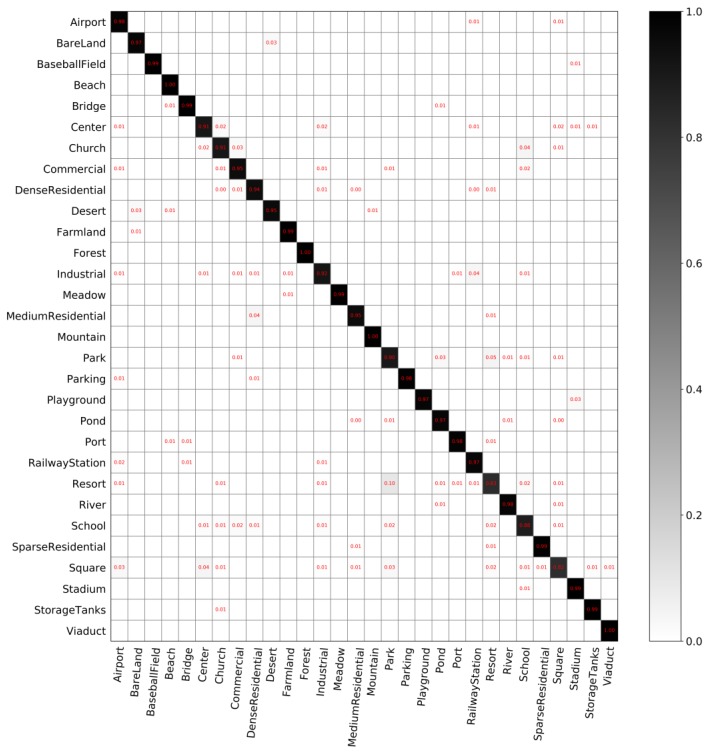
Confusion matrix on *AID* with training set of 25% and validation set of 25%.

**The classification performance of our model**. Figure 5 and Figure 6 show the confusion matrices with different partition ratios on the *AID* dataset. In these confusion matrices, each row represents the actual categories, and each column represents the predicted value. The overall accuracy in Figure 5 is 92.975%. The overall accuracy in Figure 6 is 95.86%, which is 2.885% higher than that in Figure 5.

The classification performance of our model on the *AID* dataset, shown in Figure 5 and Figure 6, leads to the following findings:

(1) Our model can classify most scenes in the *AID* dataset well. As shown in Figure 5, the classification accuracy of 23 scene categories exceeds 90%, and some categories can be exactly classified by the model, i.e., the accuracy reaches 100%. These categories include forest, meadow, and parking. In addition, the classification accuracies of some categories have reached 99%. As shown in Figure 6, the classification accuracies of 27 scene categories exceed 90%.
(2) Our model does not perform well on some scene categories. As shown in Figure 5, resort has the lowest accuracy, which is easily identified as park. Besides, the classification accuracy of squares is also relatively low because it can be easily identified as park or school. It can be found that two similar categories are easily confused.
(3) With the increasing number of training data, the classification accuracy of the model can be further improved. Compared to each category in Figure 5, the classification accuracy of each category in Figure 6 has improved, especially for category center. The classification accuracy of some categories in Figure 5 does not reach 100%, but its accuracy reaches 100% in Figure 6.

**The comparison results with some existing algorithms**. In order to prove the effectiveness of our model, we compared it to 11 algorithms. These algorithms include attention-based deep feature fusion (ADFF) [13], VGG-VD16 with DCF [47], CaffeNet with DCF [47], Multi-Branch Neural Network [30], bag of convolutional features (BOCF) [48], Scene Capture [49], late fusion local binary patterns encoded CNN models architecture (TEX-Net-LF) [50], CaffeNet [51], GoogleNet [52], VGG-16 [45], and fused global saliency-based multiscale multiresolution multistructure local binary pattern feature-local codebookless model feature (salM3LBP-CLM) [22]. The comparison results are shown in Table 3.

From Table 3, we can find that compared to ADFF, our model has a lower classification accuracy in the 20% Training Ratio experiment, but our model has a higher classification accuracy on the 50% Training Ratio experiment. It is worth mentioning that our model performs better than other algorithms except ADFF on both the 20% Training Ratio experiment and 50% Training Ratio experiment. In general, our model can classify scenes on the *AID* dataset well.

### 3.2. Experimental Results on NWPU-RESISC45 Dataset

In this section, the classification performance of our model on the *NWPU-RESISC45* dataset and the comparison results with some existing algorithms are shown.

**Figure 7:**
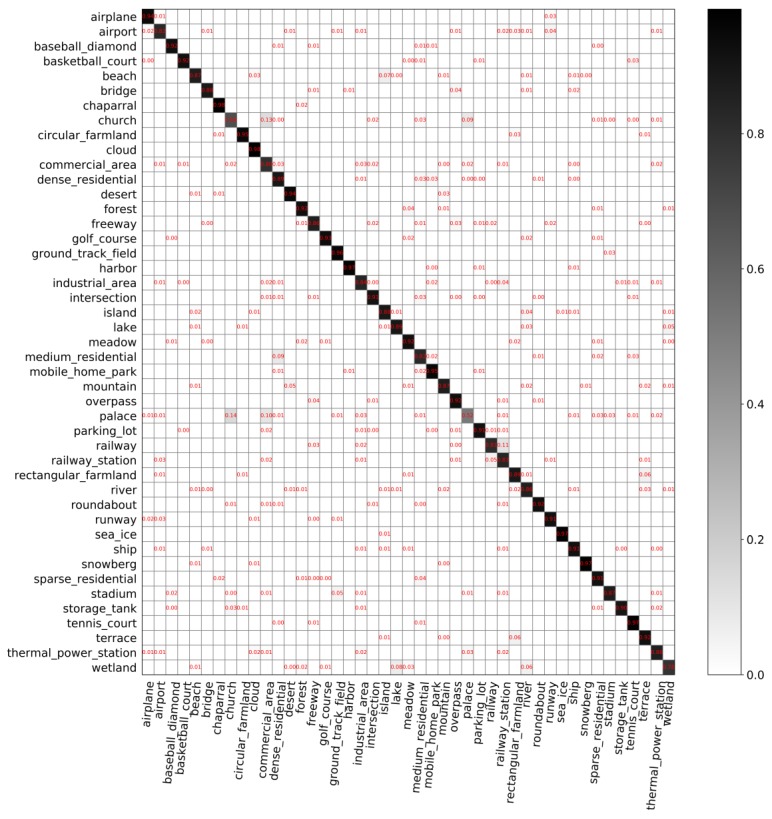
Confusion matrix on *NWPU-RESISC45* with training set of 5% and validation set of 5%.

**Figure 8:**
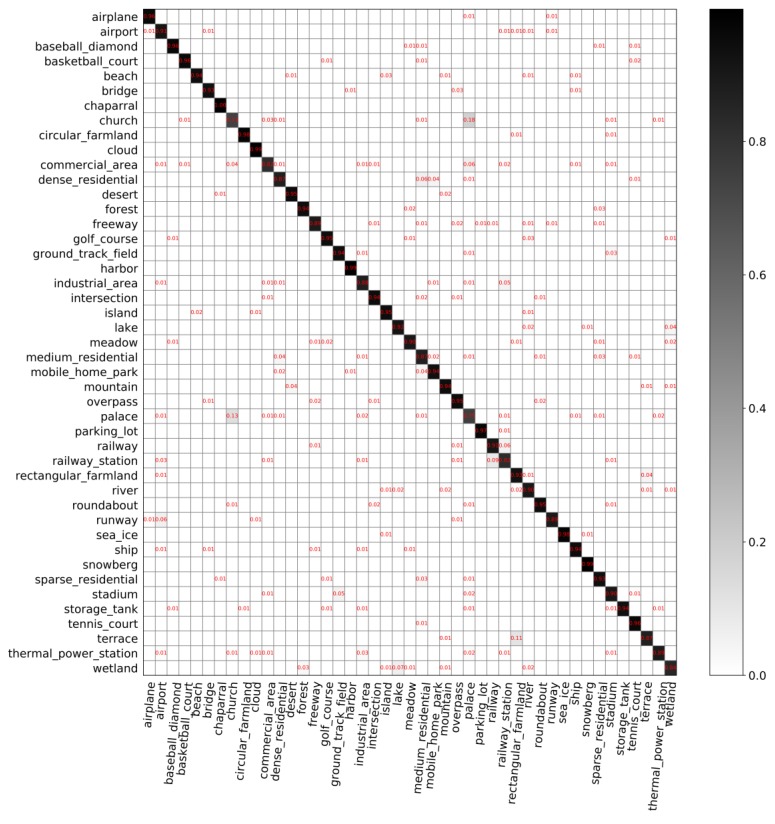
Confusion matrix on *NWPU-RESISC45* with training set of 10% and validation set of 10%.

**The classification performance of our model**. Figure 7 and Figure 8 show the confusion matrices with different partition ratios on the *NWPU-RESISC45* dataset. In those confusion matrices, each row represents the actual categories, and each column represents the predicted value. The overall accuracy in Figure 7 is 88.69%. The overall accuracy in Figure 8 is 91.92%, which is 3.23% higher than that in Figure 7.

The classification performance of our model on the *NWPU-RESISC45* dataset, shown in Figure 7 and Figure 8, leads to the following findings:

(1) The overall accuracy of the model on *NWPU-RESISC45* is lower than that on *AID*. Compared to *AID*, *NWPU-RESISC45* has more images and categories, which increases the difficulty of the dataset to some extent. In Figure 7 and Figure 8, most images are used for testing, and a small number of images are used for training. Therefore, the model performs relatively poor on this dataset.
(2) For the *NWPU-RESISC45* dataset, intra-class diversity and inter-class similarity are more obvious. From Figure 7, we can find that the classification accuracy of all categories has not reached 100% and the worst is the classification accuracy of palace, which is 52%. We can also find that the probability of palace being identified as church is 14% and being identified as commercial area is 10%. As we know, the three categories are extremely similar.
(3) (3) With the increasing number of training data, the classification performance of the model improves. As can be seen from Figure 8, the classification accuracy of palace is 75%, which is 23% higher than that shown in Figure 7. It is worth mentioning that the probability of the palace being identified as a commercial area has dropped significantly, from 10% to 1%. In addition, the classification accuracy of the chaparral increases to 100%, which is the only category where the classification accuracy can reach 100%.

**The comparison results with some existing algorithms**. On the *NWPU-RESISC45* dataset, we also compare the model with 11 algorithms. These algorithms are ADFF [13], CaffeNet with DCF [47], VGG-VD16 with DCF [47], Multi-Branch Neural Network [30], Scene Capture [49], BOCF [48], TEX-Net-LF [50], CaffeNet [51], GoogleNet [52], VGG-16 [45], and salM3LBP-CLM [22]. The comparison results are shown in Table 4.

From Table 4, it can be found that our model has worse performance compared to ADFF. It should be noted that, in addition to using Resnet to extract feature maps from the image, there is a module called SFT in ADFF, which is specially used to extract features from the attention maps and consists of several stacked convolution layers. Therefore, the parameters and computing overhead of ADFF are larger than our model. From another perspective, it is easier to increase the model parameters to improve performance. Compared to other algorithms, our model is competitive, that is to say, our model is better than the other 10 algorithms except ADFF.

### 3.3. Ablation Experiment

To discuss the effectiveness of the three modules, we perform ablation experiments. The experimental results in this section are shown. In particular, the ***baseline*** indicates the model Resnet18, ***1*** indicates that only the dropout module is added on the basis of ***baseline***, ***2*** indicates that the triple loss module is added on the basis of ***1***, and ***3*** indicates that the module of training with multi-size images is added on the basis of ***2***.

#### 3.3.1. Performances on the Training Sets and Validation Sets

**Figure 9:**
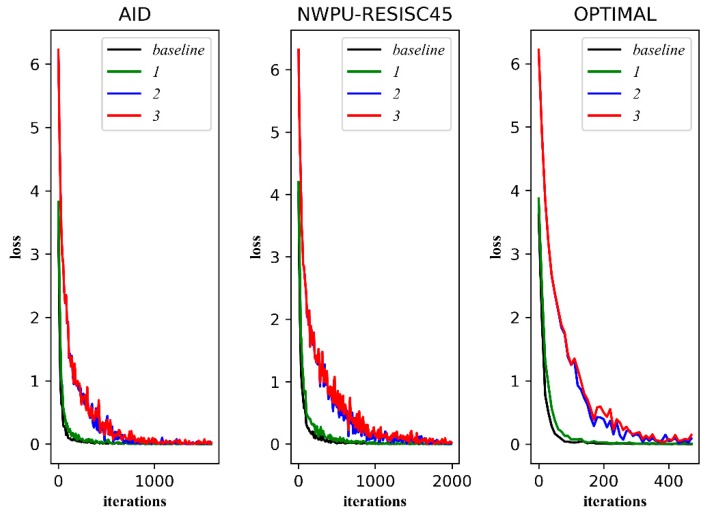
The transformation trend of the model’s loss. The horizontal axis is the number of iterations, and the vertical axis is the loss value.

**Figure 10:**
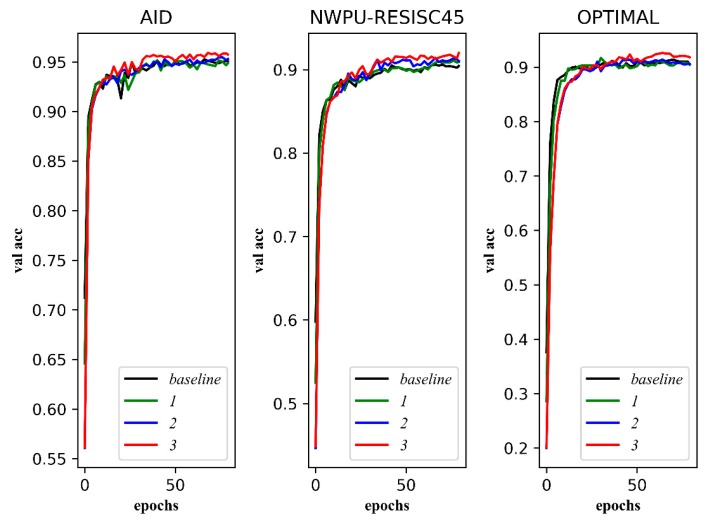
The transformation trend of the model’s overall accuracy of validation sets. The horizontal axis is the number of iterations, and the vertical axis is the overall accuracy of the Verification Set (val acc).

We visualize the transformation trend of the model on the training set and the verification set during training. Figure 9 shows the transformation trend of loss on training sets, and Figure 10 shows the transformation trend of overall accuracy on validation sets.

As shown in Figure 9, the fitting speed of ***1*** is slower than that of ***baseline***, which means that dropout makes the training speed slower. The losses of ***2*** and ***3*** are larger than those of ***1*** and ***baseline***. It can be seen that triplet loss brings more supervision information to the training. It should be noted that the loss of ***3*** fluctuates greatly, which shows that training with a multi-size image increases the complexity of the data.

As shown in Figure 10, it seems that the improvement brought by dropout and triplet loss is not obvious, but there is, in fact, still some improvement. It is worth noting that the overall accuracy of ***3*** on validation sets is the best. In other words, the gain of accuracy brought by training with multi-size images is the most obvious.

#### 3.3.2. Performances on the Validation Set and Test Set

In order to verify the effectiveness of the various modules of the algorithm, we perform ablation experiments on the *OPTIMAL* dataset. The specific partition details of the dataset are shown in Table 1. The experimental results are shown in Table 5 and “✓” indicates that the module is used.

As can be seen from Table 5, dropout, triplet loss, and training with multi-size images are all effective. First, dropout improves the generalization performance of the model. When only dropout is added, the overall accuracy of the model on the test set improves from 91.08% to 91.61%. When triplet loss is added to the model, the overall accuracy on the verification set improves from 92.12% to 92.23%, which shows that triplet loss brings better classification performance. In addition, the overall accuracy on the test set has been improved from 91.61% to 91.99%, which shows that the generalization ability of the model has also been improved. Similarly, training with multi-size images also improves the classification performance and generalization ability of the model. When it is used, the overall accuracy improves in the verification set and the test set by 0.43% and 0.7% respectively. It is worth noting that with the combined action of the three modules, the overall accuracy of the validation set improves by 0.54%, while the overall accuracy of the test set increases by 1.16%. In addition, the overall accuracy of the model on the test set is higher than that of the verification set, which also shows that the generalization ability of the model has been improved. It is worth mentioning that the addition of modules does not increase the time consumption.

#### 3.3.3. Visualization of Models

**Figure 11:**
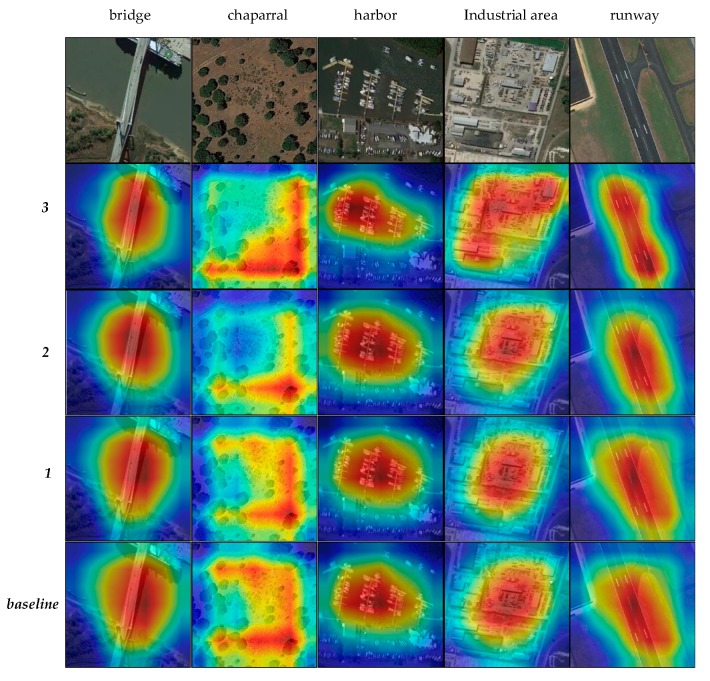
The attention maps of ***baseline***, ***1***, ***2***, and ***3***. The categories displayed are bridge, chaparral, harbor, industrial area, and runway.

According to the method provided in [32], we visualize the attention maps of ***baseline***, ***1***, ***2***, and ***3***, as shown in Figure 11.

It can be seen from Figure 11 that with the addition of modules, the model pays more attention to the target, and the shape of the attention more closely conforms to the target. It is worth mentioning that the attention of ***3*** is more regular and more fitting to the shape of the category. For example, for harbor and runway, the scope of the model perception is more accurate, more fitting to the shape of the category, while the ***baseline***, ***1***, and ***2*** focusing on the image are more rounded and the center of gravity is more diffuse.

## 4. Conclusions

In this paper, we take Resnet18 as an example to explore how to improve the classification accuracy of the model without adding other modules. The adaptive pooling makes the model process images with different sizes, enabling multi-size image training. We design a scheme of training with multi-size images to train a network. Specifically, we train the model with images of size 224 × 224 for 10 epochs. Then, the model is trained with images of size 256 × 256 for 10 epochs. After that, we train the model with images of size 288 × 288 for 10 epochs. Finally, the model is trained with images of size 320 × 320 for 50 epochs. The performance of this strategy is higher than that of a single size strategy. The branch dedicated to triplet loss can better guide the learning of the model, which makes the whole model more supervised by loss; thus, the learned features are more discriminative. Dropout embedded between the feature extractor and classifier improves the generalization ability of model. It is worth mentioning that training with multi-size images brings a greater gain in accuracy. On the *OPTIMAL* dataset, the overall accuracy of the verification set improves by 0.54%, while the overall accuracy of the test set improves by 1.61%. In general, training with multi-size images, triplet loss, and dropout are effective.

## Figures and Tables

**Table 1:** Information about datasets.

Datasets	Total Images	Scene Classes	Size	Url
*AID*	10,000	30	600 × 600	https://captain-whu.github.io/AID/
*NWPU-RESISC45*	31,500	45	256 × 256	http://www.escience.cn/people/JunweiHan/NWPU-RESISC45.html
*OPTIMAL-31*	1860	31	256 × 256	https://1drv.ms/u/s!Ags4cxbCq3lUguxW3bq0D0wbm1zCDQ

**Table 2:** Partition ratio of the datasets.

Dataset	Training Set (%)	Validation Set (%)	Test Set (%)
*AID*	10	10	80
*AID*	25	25	50
*NWPU-RESISC45*	5	5	90
*NWPU-RESISC45*	10	10	80
*OPTIMAL*	10	10	80

**Table 3:** The overall accuracy (OA) of the models on *AID*.

Methods	20% Training Ratio	50% Training Ratio	Published Year
**Ours**	**92.65 ± 0.29**	**95.456 ± 0.24**	**2019**
ADFF	93.68 ± 0.29	94.75 ± 0.24	2019
VGG-VD16 with DCF	91.57 ± 0.10	93.65 ± 0.18	2018
CaffeNet with DCF	91.35 ± 0.23	93.10 ± 0.27	2018
Multi-Branch Neural Network	89.38 ± 0.32	91.46 ± 0.44	2018
BOCF	85.24 ± 0.33	87.63 ± 0.41	2018
Scene Capture	87.25 ± 0.31	89.43 ± 0.33	2018
TEX-Net-LF	90.87 ± 0.11	92.96 ± 0.18	2017
CaffeNet	86.46 ± 0.47	89.53 ± 0.31	2017
GoogleNet	85.44 ± 0.40	88.39 ± 0.55	2017
VGG-16	86.59 ± 0.29	89.64 ± 0.36	2017
salM3LBP-CLM	86.92 ± 0.35	89.76 ± 0.45	2017

**Table 4:** The OA of the models on *NWPU-RESISC45*.

Methods	10% Training Ratio	20% Training Ratio	Published Year
**Ours**	**88.30 ± 0.24**	**91.62 ± 0.35**	**2019**
ADFF	90.58 ± 0.19	91.91 ± 0.23	2019
CaffeNet with DCF	87.59 ± 0.22	89.20 ± 0.27	2018
VGG-VD16 with DCF	87.14 ± 0.19	89.56 ± 0.25	2018
Multi-Branch Neural Network	74.45 ± 0.26	76.38 ± 0.34	2018
Scene Capture	84.84 ± 0.26	86.24 ± 0.36	2018
BOCF	83.65 ± 0.31	85.32 ± 0.17	2018
TEX-Net-LF	86.05 ± 0.24	88.37 ± 0.32	2017
CaffeNet	77.69 ± 0.21	79.85 ± 0.13	2017
GoogleNet	77.19 ± 0.38	78.48 ± 0.26	2017
VGG-16	77.47 ± 0.18	79.79 ± 0.15	2017
salM3LBP-CLM	85.32 ± 0.17	86.59 ± 0.28	2017

**Table 5:** The results of ablation experiments.

Dropout	Triplet Loss	Training with Multi-Size Images	Time on Test Set (One Image)	The OA on Validation Set (%)	The OA on Test Set (%)
			0.006s	92.12%	91.08%
**✓**			0.006s	92.12%	91.61%
**✓**	**✓**		0.005s	92.23%	91.99%
**✓**	**✓**	**✓**	0.005s	**92.66%**	**92.69%**
